# When externally oriented motivation appears to matter: shared and unique associations with creative-process practices and learning engagement among Chinese art students

**DOI:** 10.3389/fpsyg.2026.1977647

**Published:** 2026-10-21

**Authors:** Nuo Han, Zijia Xu, Yan Li

**Affiliations:** 1 Center for Teacher Education Research, Beijing Normal University, Beijing, China; 2 Dalian Polytechnic University, School of Art and Design, Dalian, Liaoning, China

**Keywords:** art education, creative-process practices, externally oriented motivation, higher education, intrinsic motivation, learning engagement

## Abstract

**Introduction:**

Motivation is closely related to students' engagement in learning, yet the associations of intrinsic motivation (IM) and externally oriented motivation (EM) may differ depending on whether they are examined separately or simultaneously. This study examined their shared and unique associations with self-reported creative-process practices (CPP) and learning engagement (LE) among Chinese university art students.

**Methods:**

Survey data were collected from 481 students enrolled in art programs in one Chinese province using purposive sampling. Confirmatory factor analysis was used to evaluate the four-factor measurement model, followed by structural equation modeling of three specifications: an IM-only model, an EM-only model, and a simultaneous IM-EM model.

**Results:**

When examined separately, both IM and EM were positively associated with CPP and LE and showed significant indirect associations with LE through CPP. When modeled simultaneously, IM retained significant associations with CPP and LE, whereas the corresponding EM associations and its indirect association through CPP were smaller and no longer statistically significant. The CPP-LE association remained positive and statistically significant across all three model specifications.

**Discussion:**

The findings indicate that externally oriented motivation can show meaningful associations when considered independently, but much of this association may overlap with intrinsic motivational variance. They also highlight the importance of distinguishing separate from unique motivational associations and of treating creative-process practices and learning engagement as related but conceptually distinct aspects of creative learning.

## 1 Introduction

Student engagement remains a central concern in higher education because it is closely related to the quality of students' learning experiences, persistence, and academic development. Engagement, however, is not simply a matter of whether students attend classes or complete assigned tasks. It also concerns the extent to which students invest energy, commitment, and psychological involvement in learning (Fredricks et al., 2004; Kahu and Nelson, 2018). Recent research has continued to demonstrate the close relationship between students' motivational orientations and their engagement in higher education, including longitudinal evidence from Chinese university students (Yang et al., 2026). Yet the ways in which different forms of motivation are associated with engagement may vary considerably across learning environments and according to how motivational constructs are conceptualized and modeled.

Art education provides a particularly informative context in which to examine these relationships. Studio-based learning typically requires students to work with problems that do not have predetermined solutions, develop and revise ideas over extended periods, experiment with materials or techniques, respond to critique, and make decisions about the direction of their own work. Systematic reviews of art studio pedagogy have characterized such learning as open-ended, student-centered, and strongly oriented toward the iterative process of making and reflection (Sawyer, 2017). Research on creative learning environments similarly emphasizes experimentation, learner agency, flexible problem solving, dialogue, and revision as important features of creative educational practice (Davies et al., 2013; Cremin and Chappell, 2021). These characteristics are not exclusive to art education, but they make the motivational conditions underlying sustained participation particularly visible. Students must often continue working despite uncertainty, incomplete solutions, unsuccessful attempts, and repeated evaluation of their developing work.

At the same time, art students rarely participate for one reason alone. Artistic learning may be intrinsically rewarding because students enjoy creating, exploring ideas, or developing personally meaningful forms of expression. It is also embedded in external educational and professional structures involving grades, portfolio requirements, exhibitions, competitions, recognition, and career preparation. Self-determination theory (SDT) provides a useful basis for understanding this coexistence because it distinguishes intrinsic motivation from extrinsic motivation while also emphasizing that externally oriented motivation is heterogeneous and varies in its degree of internalization (Ryan and Deci, 2020). Meta-analytic evidence likewise suggests that intrinsic motivation and extrinsic incentives should not automatically be treated as mutually exclusive forces; both can be associated with performance, although their relative importance depends on the nature of the outcome and the motivational context (Cerasoli et al., 2014). More broadly, research on self-determined motivation shows that different motivational regulations can coexist within the same individual and that their adaptive implications vary substantially according to motivational quality (Howard et al., 2017).

This creates an important interpretive problem. When motivational orientations are strongly related, an association observed for EM alone may partly reflect variance it shares with IM. Separate and simultaneous models therefore provide different information: the former describe associations when a motivation is considered independently, whereas the latter estimate associations unique to each motivation after their overlap is taken into account. Whether the resulting coefficients differ materially is an empirical question.

A second issue concerns what occurs between motivation and students' psychological engagement in learning. Creativity research has increasingly moved beyond treating creativity solely as the production of highly original or valuable products and has emphasized the processes through which creative work develops, including idea generation, exploration, experimentation, evaluation, and iterative refinement (Amabile and Pratt, 2016). These processes are highly relevant to studio learning, yet they should not be equated with either creative ability or learning engagement. In the present study, self-reported creative-process practices (CPP) refer to the reported frequency with which students engage in activities such as developing ideas, experimenting with materials or techniques, exploring alternative approaches, and revising their work. CPP captures participation in the creative process rather than directly observed behavior or creative product quality.

Learning engagement is conceptually distinct from CPP. In the present study, CPP refers to the reported frequency of students' participation in creative-process practices, whereas learning engagement refers to a positive psychological state characterized by vigor, dedication, and absorption (Schaufeli et al., 2002). The study therefore examines whether these two aspects of creative learning are empirically associated without treating them as interchangeable.

These questions are particularly relevant in the Chinese higher-education context. Recent research has demonstrated meaningful relationships among motivation, engagement, and learning outcomes among Chinese undergraduates (Yang et al., 2026), while research with Chinese visual-arts students has also highlighted the importance of psychological and social factors in students' creative development (Luo et al., 2023). Nevertheless, a more specific gap remains. It is unclear whether broad externally oriented motivation shows associations with creative-process practices and learning engagement that remain distinguishable once its overlap with intrinsic motivation is taken into account. It is also unclear whether the association between creative-process practices and psychological engagement remains similar when alternative motivational specifications are considered. These questions have received particularly limited attention among university art students in China. This issue matters because externally oriented goals such as exhibitions, portfolio development, recognition, assessment, and employment preparation may be both institutionally salient and personally meaningful. Treating intrinsic and extrinsic motivation as simple opposites therefore provides an incomplete account of how motivation may operate in this educational context.

The present study addresses these gaps by examining intrinsic motivation, broad externally oriented motivation, self-reported creative-process practices, and learning engagement among Chinese university art students under separate and simultaneous motivational model specifications. Rather than focusing only on whether particular paths are statistically significant, the study distinguishes among zero-order associations, associations observed when each motivational orientation is modeled separately, and the unique associations that remain when intrinsic and externally oriented motivation are modeled simultaneously. It also examines whether the association between creative-process practices and learning engagement remains comparatively stable across these alternative motivational specifications. In doing so, the study seeks to make three contributions. First, it provides a more differentiated account of motivational associations by separating relationships observed in isolation from unique relationships estimated in a joint model. Second, it clarifies the conceptual and empirical distinction between students' self-reported participation in creative processes and their psychological engagement in learning. Third, it uses art education as a theoretically informative context for examining how internally grounded interest and externally oriented goals may coexist within creative learning. Because the data are cross-sectional and self-reported, the study interprets the resulting paths and indirect effects as statistical associations rather than as evidence of temporal or causal mechanisms.

## 2 Literature review

### 2.1 Learning motivation: from motivational quantity to motivational quality

Motivation has long been regarded as a central antecedent of students' participation, persistence, and achievement. Earlier approaches often treated motivation primarily in quantitative terms, asking how much motivation a student possessed. Self-determination theory (SDT), by contrast, emphasizes that the quality and underlying reasons for motivation are as important as its intensity (Deci and Ryan, 2000; Ryan and Deci, 2000, 2020). From this perspective, students may engage in the same academic activity for substantially different reasons, and these reasons can have different implications for how they experience and sustain their involvement.

Intrinsic motivation refers to engagement in an activity because the activity itself is experienced as interesting, enjoyable, or satisfying. In educational settings, intrinsically motivated students participate because learning, exploration, or mastery is rewarding in itself rather than because participation is merely instrumental to another outcome (Ryan and Deci, 2020). A large body of research has linked more autonomous forms of motivation to persistence, deeper learning, wellbeing, and engagement. Meta-analytic evidence likewise indicates that intrinsic motivation is consistently associated with performance, particularly when the quality of performance is important rather than merely the quantity of output (Cerasoli et al., 2014). Extrinsic motivation is more complex. It refers broadly to participation directed toward outcomes separable from the activity itself, but SDT does not regard all extrinsic motivation as psychologically equivalent. External regulation is characterized by relatively direct external demands or rewards, whereas introjected regulation involves internally experienced pressures such as guilt, self-worth contingencies, or the desire to demonstrate competence. Identified regulation occurs when individuals recognize the personal importance of an activity, and integrated regulation reflects an even greater incorporation of the activity into one's values and sense of self (Deci and Ryan, 2000; Ryan and Deci, 2020). Thus, an externally structured goal can vary considerably in the extent to which it is personally endorsed.

The internalization of external goals is closely related to the satisfaction of the basic psychological needs for autonomy, competence, and relatedness. Autonomy concerns experiencing one's actions as volitional and self-endorsed; competence concerns feeling effective in interactions with the environment; and relatedness concerns experiencing meaningful connection and belonging with others. Contexts that support these needs can facilitate both intrinsic motivation and the internalization of externally initiated goals (Vansteenkiste et al., 2020). In educational settings, autonomy-supportive teaching, clear structure, and opportunities for meaningful participation have therefore been associated with stronger student engagement (Jang et al., 2010; Reeve and Cheon, 2021).

This theoretical distinction is important because extrinsic and intrinsic motivation should not be treated as simple opposites. Students can simultaneously enjoy an activity and value external outcomes associated with it. Person-oriented research has identified student profiles characterized by relatively high levels of both autonomous and controlled motivation, illustrating that different motivational orientations can coexist within the same individual rather than occupying opposite ends of a single empirical dimension (Ratelle et al., 2007). Similarly, Cerasoli et al. (2014) showed that intrinsic motivation and extrinsic incentives can jointly predict performance, although their relative importance depends on the type of outcome being considered.

Meta-analytic research indicates that motivational regulations differ systematically in their associations with educational outcomes, with more autonomous forms generally showing more adaptive patterns than more controlled forms (Howard et al., 2017). These distinctions, however, depend on measuring specific forms of regulation. The EM scale used here combines achievement- and recognition-oriented goals without distinguishing external, introjected, identified, or integrated regulation. It is therefore treated as broad externally oriented motivation rather than controlled motivation. This measurement breadth also means that an observed EM-outcome association may reflect a mixture of externally oriented goals, their degree of personal endorsement, and motivational variance shared with intrinsic interest.

### 2.2 Creative-process practices in art learning

Creativity has been conceptualized in multiple ways, including as an individual characteristic, a cognitive capacity, a creative process, a form of everyday activity, or the production of original and effective outcomes. The widely cited standard definition of creativity emphasizes the production of something both original and effective (Runco and Jaeger, 2012), whereas developmental approaches have broadened attention toward forms of creativity that occur during learning. For example, the Four-C framework distinguishes eminent creativity from professional, everyday, and personally meaningful forms of creative development (Kaufman and Beghetto, 2009). Beghetto (2016) account of creative learning similarly emphasizes the processes through which learners develop new understandings and possibilities rather than restricting creativity to exceptional products.

This distinction between creative outcome and creative process is especially consequential in educational research. A student can engage intensively in experimentation, generate multiple possibilities, revise repeatedly, and reflect critically without necessarily producing a highly original final product. Conversely, an apparently successful product does not reveal the processes through which it was produced. Process-oriented accounts of creativity therefore direct attention toward idea generation, experimentation, evaluation, revision, and elaboration as components of creative activity (Amabile and Pratt, 2016).

Art education makes these processes particularly visible because studio pedagogy is often organized around iterative making. Sawyer (2017) systematic review of 65 empirical studies of art studio teaching characterized studio pedagogy as active, constructivist, participatory, and strongly oriented toward engagement in the creative process. Students typically develop ideas through cycles of making, receiving feedback, reconsidering decisions, and producing revised iterations rather than moving directly from instruction to a predetermined correct answer. Research on studio learning similarly emphasizes the importance of hands-on participation, critique, reflective dialogue, and the progressive development of work through practice (Lynas et al., 2013). Broader research on creative learning environments has identified related pedagogical features. Davies et al. (2013), in a systematic review of creative learning environments, highlighted flexible use of space and time, appropriate use of resources, opportunities for collaboration, learner agency, and relationships characterized by mutual respect. Cremin and Chappell (2021) later systematic review of creative pedagogies likewise emphasized learner agency, possibility thinking, collaboration, problem solving, and supportive teacher-student relationships. These studies suggest that creative learning is not reducible to the final evaluation of originality; it is also expressed through the ways students explore alternatives and work through uncertainty.

The present study uses the term self-reported creative-process practices (CPP) to describe this process-oriented dimension of students‘ learning. The label is deliberately narrower than “creativity” and more cautious than “creative behavior.” CPP refers to students' reported frequency of engaging in practices such as generating and developing ideas, experimenting with materials or techniques, combining different approaches, recording emerging ideas, seeking inspiration, reflecting on work, and revising earlier solutions.

Three boundaries are particularly important. First, CPP does not assess creative product quality. The scale does not determine whether a student's artwork, design, portfolio, or other output is original, effective, aesthetically successful, or professionally valuable. Such claims would require direct assessment of products, potentially through expert judgment or other product-based creativity measures. Second, CPP is a self-reported rather than directly observed behavioral measure. Although participants reported how frequently they had engaged in specific creative-process practices during the previous academic semester, these responses remain retrospective self-reports and should not be interpreted as objective records of behavior. Third, CPP should not be equated with learning engagement. Experimenting with materials, revising an idea, or exploring alternative approaches describes what students report doing during creative work. These activities may occur with different levels of enthusiasm, energy, or psychological immersion. The distinction is therefore not merely semantic. It separates a set of reported creative-process practices from the psychological quality of students' involvement in learning.

These conceptual boundaries respond to a persistent issue in creativity research: different measures frequently capture different aspects of creativity, making broad claims difficult when the measurement target is not specified precisely. In the present study, the focus is deliberately modest. CPP is used as a process-oriented educational construct that reflects how students report approaching creative work, without treating these reports as direct evidence of either creative ability or creative achievement.

### 2.3 Learning engagement as a psychological state

Student engagement is itself a concept with multiple traditions. Some frameworks define engagement broadly in terms of behavioral, emotional, and cognitive dimensions (Fredricks et al., 2004). Behavioral engagement may include effort, persistence, attendance, and participation; emotional engagement concerns affective reactions such as interest, enjoyment, or boredom; and cognitive engagement concerns strategic investment and self-regulated learning. Later work has also proposed agentic engagement, referring to students' constructive contribution to the learning environment and instructional process (Reeve and Tseng, 2011).

In higher education, this conceptual diversity has produced substantial variation in what researchers mean by engagement. Kahu (2013) distinguished behavioral, psychological, sociocultural, and holistic perspectives and warned against conflating the state of engagement with its antecedents or outcomes. Kahu and Nelson (2018) subsequently emphasized the educational interface between students and institutions, showing that engagement emerges from interactions among individual and contextual factors rather than representing a purely stable personal characteristic. The present study adopts a more specific psychological conceptualization. Learning engagement is assessed through the framework underlying the Utrecht Work Engagement Scale and its short form, which conceptualize engagement in terms of vigor, dedication, and absorption (Schaufeli et al., 2002, 2006). Vigor refers to energy and persistence; dedication refers to involvement, significance, and enthusiasm; and absorption describes deep concentration and immersion in an activity.

This definition should not be confused with the full multidimensional student-engagement literature. The measure does not independently operationalize behavioral, emotional, cognitive, and agentic engagement; instead, it captures a positive psychological state during learning. This narrower definition is important for the present model because it keeps LE distinct from CPP. CPP captures students' reported creative-process practices, whereas LE captures the psychological quality of their involvement. The two constructs may therefore be empirically related without being definitionally identical.

Motivational research provides a strong basis for expecting engagement to be responsive to students‘ reasons for participating. SDT perspectives describe engagement as one pathway through which motivational experiences become visible in learning activity (Reeve, 2012). Empirical work also shows that autonomy support and instructional structure are associated with stronger engagement, while students' own motivational states contribute to the quality of their participation (Jang et al., 2010). Theoretical and empirical research thus positions engagement as a dynamic psychological state closely connected to motivation and learning conditions, rather than as a simple synonym for participation.

For the present study, this distinction helps establish a three-level conceptual structure: motivation concerns why students are oriented toward learning; CPP concerns how they report approaching the creative process; and learning engagement concerns how psychologically energized, dedicated, and absorbed they feel during learning. These constructs may be strongly related, but their conceptual roles remain different.

### 2.4 Art education as a motivational context: international and Chinese evidence

The relevance of motivation and creative-process practices needs to be considered in relation to the educational context in which they occur. Art education should not be portrayed as wholly unique; experimentation, revision, uncertainty, and project-based work also occur in engineering, laboratory sciences, architecture, and other practice-oriented disciplines. Nevertheless, studio-based art education provides a theoretically revealing setting because creative development, iterative making, critique, and evaluation are often central rather than peripheral to the curriculum.

Studio pedagogy typically places students in an ongoing relationship with both personal and external criteria. Students are encouraged to develop individual approaches, explore possible solutions, and construct artistic or design meaning, while simultaneously working within assessment systems, briefs, deadlines, critique sessions, portfolio expectations, exhibitions, and professional standards. This combination creates conditions in which internally grounded interest and externally oriented goals can coexist within the same learning activity. Such coexistence is particularly important when considering creativity. Creative work often requires persistence through uncertain outcomes, tolerance for incomplete solutions, and willingness to revise unsuccessful attempts. Intrinsic interest may support this persistence because exploration is experienced as worthwhile in itself. At the same time, external goals such as completing a portfolio, gaining recognition, preparing for employment, or succeeding in an exhibition can provide powerful reasons for effort. Their psychological significance, however, depends partly on whether students experience them as controlling demands or as meaningful goals incorporated into their own artistic development.

Against this international background, research on Chinese art education underscores the need for contextual caution. Saris (2020), in a review of creativity and creative-design-process learning in visual communication design in China, noted both the limited empirical base and the difficulty of transferring Western definitions and models of creativity into Chinese educational contexts without examining cultural and institutional conditions. Rather than assuming that Chinese students approach creative learning in a uniform way, this literature calls for more context-sensitive examination of how learners engage with creative processes. Recent empirical research has begun to expand this evidence base. Luo et al. (2023), studying 1,198 Chinese visual-arts college students, demonstrated that students' understandings of creativity and creative self-efficacy are shaped by social and gendered beliefs, illustrating that creativity-related learning is embedded in broader cultural meanings rather than functioning as a context-free individual trait. More recent work with Chinese art undergraduates has also examined the role of reflective practice, self-regulated learning, learning environment, and other psychological characteristics in creative development (Jianhao et al., 2026). These studies point toward a growing interest in the processes through which students learn creatively, rather than focusing only on final creative outcomes.

Motivation is also receiving greater attention in this population. Wu and Wang (2026), using a mixed-methods design with Chinese art undergraduates, found that several forms of learning motivation were related to critical thinking and that intrinsic motivation showed a particularly strong positive relationship. Their qualitative findings additionally highlighted autonomy, competence, relatedness, feedback, collaboration, and iterative revision as important features of students' learning experiences. Although the outcome in that study was critical thinking rather than learning engagement, the findings reinforce the relevance of motivational quality and basic psychological needs within contemporary Chinese art education.

At the same time, existing research leaves several issues unresolved. Studies frequently examine motivation, creativity-related variables, or engagement independently, but relatively little work has explicitly separated students' creative-process practices from their psychological engagement while simultaneously considering different motivational orientations. Furthermore, positive associations involving external goals are often difficult to interpret when intrinsic and externally oriented motivation overlap substantially. Without distinguishing the associations observed when each orientation is considered separately from those that remain when both are modeled together, it is difficult to determine whether the apparent contribution of externally oriented motivation represents a unique relationship or variance shared with broader motivational involvement.

The literature therefore supports a more differentiated analysis of motivation in art education. Rather than asking only whether intrinsic and extrinsic motivation are positively related to learning, it is necessary to consider what form of extrinsic motivation has actually been measured, how motivational orientations coexist, whether creative-process practices are distinct from psychological engagement, and how the observed relationships change under different model specifications. These issues provide the basis for the theoretical analysis developed in the following section.

## 3 Theoretical analysis and hypotheses

### 3.1 Intrinsic motivation in relation to creative-process practices and learning engagement

Intrinsic motivation provides a theoretically strong starting point for understanding students' involvement in open-ended learning. Building on the SDT account reviewed above (Ryan and Deci, 2000, 2020), its relevance lies not simply in the amount of effort students expend, but in directing attention toward the activity itself. When learning and exploration are experienced as rewarding in themselves, continued participation can remain worthwhile even when immediate external rewards are absent.

This distinction is especially relevant to creative-process practices. Creative work commonly involves incomplete ideas, uncertain outcomes, unsuccessful attempts, and repeated revision. The dynamic componential model of creativity identifies intrinsic task motivation as an important condition for sustained engagement with creative problems because interest in the task can support exploration beyond minimally acceptable solutions (Amabile and Pratt, 2016). In educational settings, creative learning similarly requires learners to generate possibilities, tolerate uncertainty, reconsider prior assumptions, and remain open to alternative approaches (Beghetto, 2016; Cremin and Chappell, 2021).

In art education, these demands are embedded in the ordinary structure of studio work. Students may need to test alternative materials, reconsider an initial concept after critique, abandon unsuccessful directions, or continue developing an idea without knowing whether the final work will meet assessment or professional expectations. Sawyer (2017) review of studio pedagogy emphasizes that creative learning in art develops through iterative making, reflection, feedback, and revision rather than through the reproduction of fixed solutions. Under such conditions, intrinsic motivation should be particularly relevant to students' willingness to participate in the creative process itself. If experimenting, exploring, and developing artistic ideas are experienced as inherently worthwhile, students have a reason to continue these practices even when immediate success is uncertain. The proposed relationship concerns students' participation in creative-process activities rather than the originality or quality of their final creative products.

Intrinsic motivation should also be positively associated with learning engagement. From an SDT perspective, students are more likely to become energetically and persistently involved in activities that they experience as self-endorsed and personally meaningful (Reeve, 2012; Ryan and Deci, 2020). Empirical research has repeatedly associated autonomous motivation with adaptive forms of academic engagement and persistence, while educational contexts that support autonomy and competence tend to facilitate more active involvement in learning (Jang et al., 2010; Reeve and Cheon, 2021).

The psychological-state definition of engagement adopted in the present study makes this association theoretically coherent. Vigor concerns the energy students experience while learning; dedication concerns enthusiasm, significance, and commitment; and absorption concerns deep concentration in the activity (Schaufeli et al., 2002). Students who learn art or design because they genuinely enjoy developing ideas and understanding artistic problems should, on average, be more likely to report these psychological qualities than students whose participation is less internally grounded.

More directly, research with fine-arts students has shown that experimentally induced intrinsic motivation was associated with more positive affect and more favorable self-evaluation across successive stages of the creative process, providing adjacent empirical evidence that intrinsic motivation is relevant to students' experience of creative activity rather than only to final creative products (Stanko-Kaczmarek, 2012). Recent research in Chinese art education also points toward the importance of internally grounded motivation. Wu and Wang (2026), for example, found that intrinsic motivation showed a particularly strong positive relationship with critical thinking among Chinese art undergraduates, while qualitative evidence highlighted autonomy, competence, and relatedness as meaningful features of students' learning experiences. Although critical thinking differs from learning engagement, this evidence is consistent with the broader proposition that intrinsically grounded motivation is associated with adaptive forms of participation in creative higher education. Direct evidence linking intrinsic motivation specifically to the present CPP measure remains limited, so H1a extends these theoretical and adjacent empirical findings to self-reported creative-process practices in university art learning. Accordingly:

H1a. Intrinsic motivation is positively associated with self-reported creative-process practices.

H1b. Intrinsic motivation is positively associated with learning engagement.

### 3.2 Creative-process practices and learning engagement

CPP and learning engagement are conceptually distinct, but there are strong reasons to expect them to be positively associated. CPP captures students' reported participation in processes such as developing ideas, experimenting with materials or techniques, exploring alternatives, reflecting on emerging work, and revising earlier decisions. Learning engagement, in contrast, concerns the psychological experience of vigor, dedication, and absorption. The theoretical question is therefore not whether creative-process activity is itself engagement, but whether greater participation in such practices tends to accompany a more engaged psychological state.

Participation in creative-process practices involves students actively shaping aspects of their own learning. Generating alternatives, experimenting with materials or techniques, and revising earlier work require choice, interpretation of uncertain outcomes, and evaluative judgment. These practices create opportunities for choice, judgment, feedback, and iterative problem solving, all of which can make learning more personally consequential.

This reasoning is consistent with research on creative learning environments. Davies et al. (2013) identified learner agency, flexible approaches to tasks, collaboration, and opportunities for experimentation as recurring features of environments that support creativity. Cremin and Chappell (2021) similarly highlighted agency, possibility thinking, problem solving, and dialogue within creative pedagogies. In studio education specifically, learning typically occurs through cycles of making, critique, reflection, and further development (Sawyer, 2017). Recent work with Chinese art students has likewise found reflective practice to be positively associated with creativity during the learning process, reinforcing the importance of iterative engagement with developing work (Jianhao et al., 2026).

The expected CPP-engagement relationship can also be understood in motivational terms. When students participate actively in shaping a creative task, they may have greater opportunity to experience competence through problem solving and autonomy through meaningful choice. These experiences are theoretically relevant to engagement within SDT (Jang et al., 2010; Vansteenkiste et al., 2020). However, the present study does not measure psychological-need satisfaction, and therefore does not test autonomy or competence as explanatory mechanisms. They provide a theoretical account of why creative-process participation and psychological engagement may be related, not variables through which the relationship is empirically established. Direct quantitative tests of the association between process-oriented creative practices and psychological engagement among university art students remain scarce; H2 therefore extends the broader creative-learning and studio-pedagogy literature to this specific relationship. Because the data are cross-sectional, H2 concerns association rather than temporal direction; greater engagement may also accompany a greater willingness to experiment and revise. Accordingly:

H2. Self-reported creative-process practices are positively associated with learning engagement.

### 3.3 The indirect association between intrinsic motivation and learning engagement through creative-process practices

Taken together, the preceding arguments suggest a theoretically coherent indirect association in which intrinsic motivation is related to learning engagement through self-reported creative-process practices. Students who experience artistic learning as interesting or satisfying may be more willing to explore incomplete ideas, test alternatives, reconsider unsuccessful attempts, and continue revising their work. Greater participation in these practices may, in turn, accompany stronger energy, dedication, and immersion in learning.

This pattern is compatible with componential and educational accounts of creativity. Amabile and Pratt (2016) describe intrinsic task motivation as one component supporting creative activity, while creative learning research emphasizes exploration, interpretation, and iterative development as central to learners' participation in creative tasks (Beghetto, 2016). From an engagement perspective, student motivation can be expressed through the quality of students' participation in learning rather than existing only as an internal psychological disposition (Reeve, 2012). CPP can therefore be conceptualized as one process-level correlate that connects students' reasons for learning with their broader psychological involvement.

Importantly, however, the present study does not claim to establish a temporal mediation mechanism. A statistically significant indirect association in cross-sectional data cannot demonstrate that intrinsic motivation occurs first, produces CPP, and subsequently causes greater engagement. Cross-sectional mediation estimates can be biased when the underlying process unfolds over time, and alternative causal ordering cannot be excluded (Maxwell and Cole, 2007). The indirect pathway is therefore treated as an indirect statistical association consistent with the proposed theoretical ordering, rather than evidence that CPP mediates a causal process. Accordingly:

H3. Intrinsic motivation has a positive indirect association with learning engagement through self-reported creative-process practices.

### 3.4 Broad externally oriented motivation and its associations with creative-process practices and learning engagement

Theoretical expectations are less straightforward for the broad EM construct used here. Within SDT, externally oriented reasons differ substantially in their degree of internalization: pursuing an outcome to obtain a reward or avoid disapproval is psychologically different from pursuing it because the outcome has been personally valued (Deci and Ryan, 2000; Ryan and Deci, 2020).

This heterogeneity is particularly relevant to art education. Grades, exhibitions, portfolio development, competitions, recognition, and employment preparation are externally structured outcomes, but their psychological meanings cannot be assumed to be identical. A student may pursue an exhibition primarily to gain external approval. Another may value the same exhibition because it provides an opportunity to develop an artistic identity or enter a desired professional field. In both cases, the goal is separable from the immediate activity, yet the degree of personal endorsement differs.

Meta-analytic research confirms that extrinsic incentives and intrinsic motivation can coexist and that their relationships with performance depend partly on the type of incentive and the type of performance being considered (Cerasoli et al., 2014). Similarly, Howard et al. (2017) meta-analysis supports the broader SDT proposition that motivational regulations form a differentiated continuum and display different relationships with adaptive outcomes. These findings make a single directional prediction for a broad extrinsic-motivation score difficult to justify.

The measurement used in the present study reinforces this need for caution. The retained EM items concern externally oriented achievement and recognition goals, but they do not identify whether those goals reflect external regulation, introjection, identification, or integration. Consequently, the construct should not be treated as synonymous with controlled motivation. Nor is there a strong theoretical basis for assuming that it must either promote or undermine creative-process practices and engagement.

Externally oriented goals could be positively associated with CPP because portfolio development, exhibitions, recognition, or achievement may motivate students to invest substantial effort in developing and revising work. They could also be positively associated with psychological engagement when students value those outcomes. On the other hand, highly evaluative or controlling goals may encourage safer strategies, performance monitoring, or minimum-compliance behavior, potentially weakening the relationship with exploratory creative practice. The broad EM measure combines these possibilities rather than separating them. For this reason, the study treats the role of broad externally oriented motivation as an empirical question rather than imposing a directional hypothesis:

RQ1. When broad externally oriented motivation is considered separately, how is it associated with self-reported creative-process practices and learning engagement, and does it show an indirect association with learning engagement through creative-process practices?

### 3.5 Shared and unique motivational associations across alternative model specifications

A further theoretical issue concerns the distinction between an association observed when a motivational orientation is considered on its own and the unique association that remains when multiple motivational orientations are considered simultaneously. This distinction is fundamental when intrinsic and externally oriented motivation are not mutually exclusive.

SDT does not require students to possess only one motivational orientation. Individuals can participate in the same activity because it is enjoyable and because it serves valued educational or professional outcomes. Person-oriented work has identified combinations in which autonomous and controlled motivation coexist at relatively high levels, demonstrating that motivational types need not form mutually exclusive categories (Ratelle et al., 2007). Cerasoli et al. (2014) similarly showed that intrinsic motivation and extrinsic incentives can contribute jointly to performance rather than functioning as simple opposites.

The separate and simultaneous models answer different statistical questions. The separate models estimate the associations of each motivational orientation with CPP and LE when considered independently, whereas the simultaneous model estimates the association unique to each orientation after their overlap is taken into account. The direction and magnitude of any change in these coefficients are treated as empirical questions rather than specified in advance. The same logic applies to the CPP-LE path. Examining this path across alternative motivational specifications allows the study to determine whether its estimated association changes when IM, EM, or both are included in the structural model. No particular degree of stability is assumed a priori.

The present study therefore treats model comparison not as a competition to identify a single “best” motivational model, but as a means of distinguishing separate from unique associations. Because the models contain different sets of motivational indicators, the purpose is not to rank them using information criteria. Instead, the analysis examines how the substantive pattern of paths changes when motivational overlap is and is not taken into account. Accordingly:

RQ2. How do the associations of intrinsic motivation and broad externally oriented motivation with self-reported creative-process practices and learning engagement change when the two motivational orientations are modeled simultaneously rather than separately, and to what extent does the CPP-learning engagement association remain stable across these alternative model specifications?

The proposed relationships and research questions are summarized in Figure 1.

**Figure 1:**
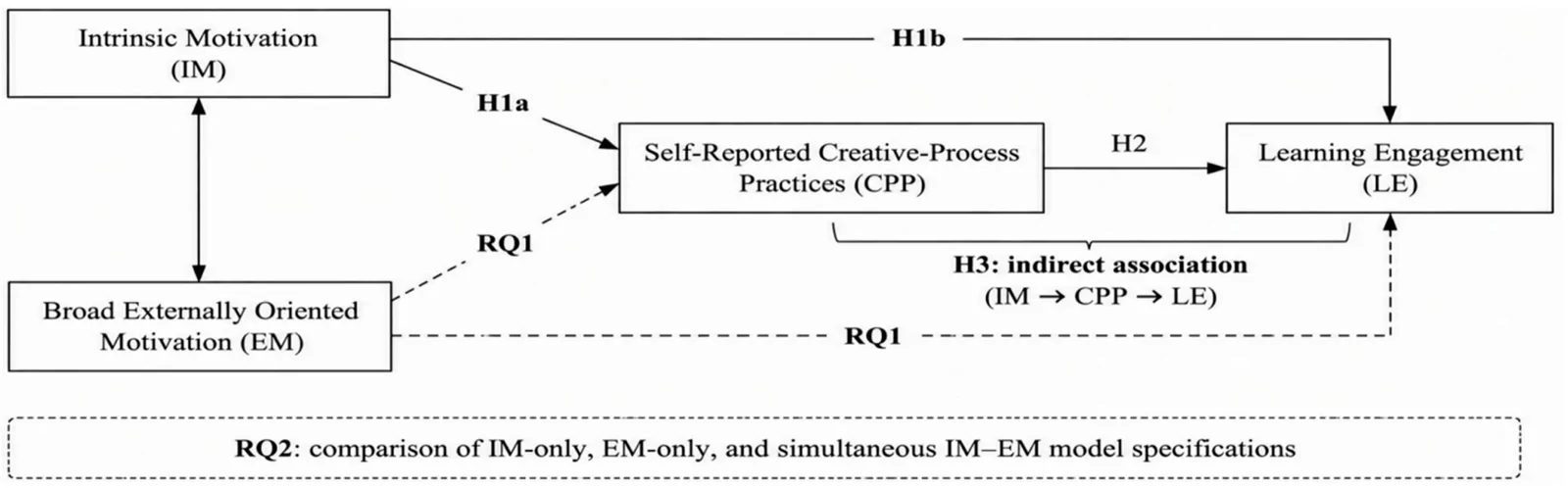
Conceptual model of the proposed relationships. Solid lines represent hypothesized paths; dashed lines represent relationships examined under RQ1. H3 refers to the indirect association IM → CPP → LE. RQ2 concerns comparisons across model specifications.

## 4 Methods

### 4.1 Participants and data collection

Participants were recruited through purposive sampling from university art programs located in one province of China. The study focused on students enrolled in art- and design-related programs because the research questions concerned motivation, creative-process practices, and learning engagement within learning environments characterized by studio work, iterative production, and creative problem solving. Data were collected through Wenjuanxing, a widely used online survey platform in China.

Prior to completing the questionnaire, participants were informed of the purpose of the study, the voluntary nature of participation, procedures for anonymity and confidentiality, and their right to withdraw at any time without penalty. Electronic informed consent was obtained before access to the survey items was granted. No directly identifying information was collected. Under the applicable institutional and national regulations, the study was exempt from formal ethics review. All procedures followed the principles of voluntary participation, informed consent, anonymity, and confidentiality.

Table 1 summarizes the sample characteristics. A total of 512 questionnaires were initially collected. Data screening was conducted before the substantive analyses. Responses were excluded when completion time was shorter than 120 s or when clear straight-lining or patterned response behavior suggested insufficient engagement with the questionnaire. After screening, 481 of the 512 submitted questionnaires were retained, corresponding to a post-screening retention rate of 93.9%. Because recruitment was conducted through participating university art programs and the total number of students who received or viewed the survey invitation was not recorded, a conventional survey response rate could not be calculated.

**Table 1:** Sample characteristics (*N* = 481).

Characteristic	Category	*n*	%
Gender	Male	251	52.2
	Female	230	47.8
Grade	First-year	155	32.2
	Second-year	120	24.9
	Third-year	90	18.7
	Fourth-year	65	13.5
	Graduate and above	51	10.6
Major	Painting	75	15.6
	Design	260	54.1
	Sculpture/Arts & Crafts	73	15.2
	Others	73	15.2
Weekly practice duration	< 5 h	196	40.7
	5-10 h	181	37.6
	11-15 h	51	10.6
	16-20 h	16	3.3
	>20 h	37	7.7

### 4.2 Measures

The questionnaire was developed by adapting established instruments in motivation, creativity, and engagement research to the context of university art education. The source items were professionally translated into Chinese and subsequently checked against the English versions to ensure conceptual consistency. A formal independent back-translation procedure was not conducted. Three experts reviewed the translated and adapted questionnaire for semantic clarity, content relevance, and contextual appropriateness, and a pilot test with 20 students was conducted before formal data collection to identify potential wording or comprehension problems.

IM and EM items were rated on five-point endorsement scales, CPP items on a five-point self-reported frequency scale ranging from 1 (never) to 5 (always), and LE items on a five-point agreement scale. Higher scores indicated higher levels of the corresponding constructs. The use of different response formats across CPP and the motivation and engagement measures may also reduce response-pattern consistency across constructs; anonymity and confidentiality were emphasized during data collection.

#### 4.2.1 Intrinsic motivation

Intrinsic motivation (IM) was assessed using 7 items adapted with reference to the Academic Motivation Scale (AMS; Vallerand et al., 1992). The items were contextualized to art learning and assessed students' interest, enjoyment, and satisfaction derived from learning and creative activity itself. IM therefore represents motivation grounded in the inherent interest or satisfaction of engaging in art learning rather than in outcomes separable from the activity, consistent with the SDT conceptualization of intrinsic motivation (Ryan and Deci, 2020).

The initial motivation item pool also included several items concerning avoidance of failure or challenging tasks. These items were not retained in the focal motivation scales because their content represented an avoidance-oriented motivational tendency that was conceptually distinct from both intrinsic motivation and the externally oriented motivation examined in this study. Their exclusion therefore reflected the conceptual scope of the study rather than an assumption that avoidance motivation lacks relevance to art learning.

#### 4.2.2 Broad externally oriented motivation

Broad externally oriented motivation (EM) was initially represented by a pool of candidate items adapted with reference to the AMS (Vallerand et al., 1992). The 4 retained items captured motivation directed toward outcomes separable from the immediate learning activity, particularly achievement- and recognition-oriented outcomes. One additional candidate item stated a preference for undertaking less demanding art projects to guarantee success. This item was initially retained because its wording referred to an outcome-oriented reason for task selection rather than enjoyment of the activity itself. However, it was retained for measurement-model evaluation rather than assumed a priori to represent the final EM construct. The final EM construct is described as broad externally oriented motivation because the retained items do not differentiate external, introjected, identified, and integrated regulation (Ryan and Deci, 2020). Accordingly, a higher EM score should not be interpreted as indicating any particular degree of motivational internalization. Further details on excluded motivation items are provided in Supplementary Table 1.

#### 4.2.3 Self-reported creative-process practices

Self-reported creative-process practices (CPP) were assessed using 12 items adapted with reference to Hocevar's Creative Behavior Inventory (CBI; Hocevar, 1979) and contextualized for art and design learning. The items addressed students' reported participation in processes such as developing ideas, experimenting with materials or techniques, exploring alternative approaches, and revising decisions during creative work. Participants rated how frequently they had engaged in these practices during the previous academic semester on a five-point scale ranging from 1 (*never*) to 5 (*always*). CPP therefore represents self-reported frequency of creative-process practices rather than directly observed behavior. This process-oriented operationalization is consistent with accounts of creative activity that emphasize idea development, experimentation, evaluation, and iterative revision (Amabile and Pratt, 2016; Sawyer, 2017).

The CBI served as an item source rather than as a direct adoption of the original creative-behavior construct. Items were selectively adapted and contextualized to describe process-oriented activities occurring within university art learning. Because the resulting measure asks students how frequently they engaged in specific practices during the previous academic semester, rather than assessing creative accomplishments, products, or creative behavior across broader life domains, the narrower label “creative-process practices” was used to more closely match the content and context of the adapted measure.

#### 4.2.4 Learning engagement

Learning engagement (LE) was assessed using 9 items adapted from the engagement framework developed by Schaufeli et al. (2002) and the nine-item short form reported by Schaufeli et al. (2006). The items were reworded to refer specifically to academic and art-learning experiences. Their content reflects vigor, dedication, and absorption, which characterize students' positive psychological involvement in academic activity. In the present study, the 9 items were modeled as a single latent LE factor rather than as three separate latent subdimensions. This treatment is consistent with recent psychometric evidence among Chinese undergraduates supporting a strong general academic-engagement factor in the UWES-S-9 (Wei et al., 2025). LE therefore refers here to a positive psychological state characterized by energy, commitment, and immersion in academic and studio-based learning. This definition is intentionally narrower than multidimensional frameworks that conceptualize student engagement in terms of behavioral, emotional, cognitive, and agentic components.

LE was modeled separately from CPP: CPP captures students' reported creative-process practices, whereas LE captures the psychological quality of their involvement in learning. The two constructs may therefore be empirically related without being treated as interchangeable at the measurement level.

### 4.3 Analytical strategy

All analyses were conducted in R. Descriptive statistics and Pearson correlations were first calculated for the four focal constructs. Confirmatory factor analysis (CFA) was then used to evaluate the four-factor measurement model comprising intrinsic motivation (IM), broad externally oriented motivation (EM), self-reported creative-process practices (CPP), and learning engagement (LE). Model fit was assessed using χ^2^/df, CFI, TLI, RMSEA, and SRMR (Hu and Bentler, 1999). Internal consistency and convergent validity were evaluated using Cronbach's α, composite reliability (CR), and average variance extracted (AVE), while discriminant validity was assessed using the Fornell-Larcker criterion (Fornell and Larcker, 1981).

Structural equation modeling (SEM) was subsequently used to test the hypotheses and research questions. Three model specifications were estimated: an IM-only model, an EM-only model, and a simultaneous model including both IM and EM. The separate models were used to examine the associations of each motivational orientation with CPP and LE when considered independently, whereas the simultaneous model was used to estimate their unique associations after accounting for shared motivational variance. The CPP-LE path was also compared across the three specifications to examine its relative stability.

Indirect associations through CPP were estimated using 5,000 bootstrap resamples with percentile-based 95% confidence intervals. Because the data were cross-sectional, these indirect associations were interpreted as statistical associations rather than evidence of temporal or causal mediation (Maxwell and Cole, 2007). The three structural specifications were used for substantive comparison of association patterns rather than for selecting a single best-fitting model.

## 5 Results

### 5.1 Measurement model

A confirmatory factor analysis (CFA) was conducted to evaluate the measurement model comprising intrinsic motivation (IM), broad externally oriented motivation (EM), self-reported creative-process practices (CPP), and learning engagement (LE). The final model contained 32 items: 7 for IM, 4 for EM, 12 for CPP, and 9 for LE. The four-factor model showed an acceptable fit to the data, χ^2^(458) = 1,509.518, χ^2^/df = 3.296, RMSEA = 0.069, 90% CI [0.065, 0.073], CFI = 0.928, TLI = 0.922, and SRMR = 0.042. All retained items loaded significantly on their intended latent constructs (p < 0.001), with standardized factor loadings ranging from 0.681 to 0.901 across the four constructs.

During measurement-model evaluation, the additional candidate EM item described above showed a standardized loading of 0.483, compared with loadings of 0.818–0.901 for the retained EM items. Its content also aligned more closely with avoidance of challenge than with the achievement- and recognition-oriented focus of the retained EM items. Given both its conceptual mismatch and comparatively weak loading, the item was removed before estimation of the structural models. The final EM measure therefore comprised 4 items.

As shown in Table 2, the four constructs demonstrated satisfactory internal consistency and convergent validity. Cronbach's α values ranged from 0.922 to 0.954, and composite reliability values ranged from 0.923 to 0.954. These high internal-consistency coefficients indicate strong item coherence, although values above 0.90 may also reflect similarity among some item contents; they were therefore interpreted together with the factor loadings, CR, AVE, and discriminant-validity evidence rather than as stand-alone evidence of measurement quality. Average variance extracted (AVE) exceeded 0.50 for all four constructs, ranging from 0.635 to 0.749. Discriminant validity was further examined using the Fornell-Larcker criterion. As shown in Table 3, the square root of AVE for each construct exceeded its corresponding interconstruct correlations, supporting the empirical distinction among IM, EM, CPP, and LE. Of particular relevance to the distinction between CPP and LE, their correlation (*r* = 0.656) was lower than the square root of AVE for both CPP (0.797) and LE (0.831).

**Table 2:** Measurement model results.

Variable	No. of items	Loading range	Cronbach's α	CR	AVE
IM	7	0.726-0.897	0.945	0.946	0.714
EM	4	0.818-0.901	0.922	0.923	0.749
CPP	12	0.681-0.866	0.954	0.954	0.635
LE	9	0.770-0.899	0.952	0.953	0.691

IM, intrinsic motivation; EM, broad externally oriented motivation; CPP, self-reported creative-process practices; LE, learning engagement; CR, composite reliability; AVE, average variance extracted.

**Table 3:** Descriptive statistics, correlations, and discriminant validity.

Variable	*M*	*SD*	1	2	3	4
IM	3.764	0.771	**0.845**			
EM	3.891	0.844	0.733^***^	**0.865**		
CPP	3.224	0.738	0.517^***^	0.443^***^	**0.797**	
LE	3.719	0.733	0.709^***^	0.605^***^	0.656^***^	**0.831**

*N* = 481. Bold diagonal values represent the square root of average variance extracted ($\sqrt{AVE}$). IM, intrinsic motivation; EM, broad externally oriented motivation; CPP, self-reported creative-process practices; LE, learning engagement. ^***^p < 0.001.

### 5.2 Descriptive statistics and zero-order associations

Table 3 presents the descriptive statistics and zero-order correlations among the study variables. All four constructs were positively correlated with one another (all *p* < 0.001). IM was correlated with EM (*r* = 0.733) and LE (*r* = 0.709), while CPP was correlated with LE (*r* = 0.656). The IM-EM correlation corresponds to approximately 53.7% shared variance at the composite-score level (r^2^ = 0.537), indicating considerable overlap between the two motivational orientations while leaving substantial nonshared variance. The similarly high IM–LE correlation represents a predictor–outcome association rather than overlap between the two motivational predictors. The simultaneous model was therefore theoretically motivated to estimate the unique associations of IM and EM after accounting for their overlap, rather than being based on a predetermined correlation threshold.

### 5.3 Structural associations across motivational model specifications

As shown in Table 4, both separate motivational models showed acceptable fit. The IM-only model yielded χ^2^/df = 3.629, RMSEA = 0.074, CFI = 0.927, TLI = 0.921, and SRMR = 0.041, whereas the EM-only model yielded χ^2^/df = 3.529, RMSEA = 0.073, CFI = 0.936, TLI = 0.929, and SRMR = 0.042. In the IM-only model, IM was positively associated with CPP (β = 0.539, *p* < 0.001) and LE (β = 0.547, *p* < 0.001), while CPP was positively associated with LE (β = 0.391, *p* < 0.001). The indirect association between IM and LE through CPP was also significant [effect = 0.197, Boot SE = 0.029, 95% CI (0.142, 0.254)]. These findings supported H1a, H1b, and H3. The positive CPP–LE association also supported H2 and remained significant across all three specifications. In the EM-only model, EM was positively associated with CPP (β = 0.458, *p* < 0.001) and LE (β = 0.416, *p* < 0.001), and CPP was positively associated with LE (β = 0.496, *p* < 0.001). The indirect association between EM and LE through CPP was also significant [effect = 0.203, Boot SE = 0.032, 95% CI (0.143, 0.267)]. Thus, in response to RQ1, broad externally oriented motivation showed positive associations with both CPP and LE when considered separately, together with a significant indirect association through CPP.

**Table 4:** Structural associations across the three motivational model specifications.

**Model**	Path	β/Effect	SE/Boot SE	*p*	95% CI
IM-only model	IM → CPP	0.539	0.035	< 0.001	-
	IM → LE	0.547	0.032	< 0.001	-
	CPP → LE	0.391	0.034	< 0.001	-
	IM → CPP → LE	0.197	0.029	< 0.001	[0.142, 0.254]
EM-only model	EM → CPP	0.458	0.039	< 0.001	-
	EM → LE	0.416	0.035	< 0.001	-
	CPP → LE	0.496	0.034	< 0.001	-
	EM → CPP → LE	0.203	0.032	< 0.001	[0.143, 0.267]
IM + EM model	IM → CPP	0.472	0.070	< 0.001	-
	EM → CPP	0.085	0.074	0.248	-
	IM → LE	0.481	0.053	< 0.001	-
	EM → LE	0.085	0.052	0.101	-
	CPP → LE	0.387	0.034	< 0.001	-
	IM → CPP → LE	0.172	0.038	< 0.001	[0.104, 0.252]
	EM → CPP → LE	0.030	0.028	0.294	[−0.028, 0.081]

β denotes standardized coefficients for direct structural paths. Indirect associations were estimated using 5,000 bootstrap resamples with percentile-based 95% confidence intervals. IM, intrinsic motivation; EM, broad externally oriented motivation; CPP, self-reported creative-process practices; LE, learning engagement.

The pattern changed when IM and EM were modeled simultaneously. As shown in Figure 2, the simultaneous model showed acceptable fit, χ^2^(458) = 1509.518, χ^2^/df = 3.296, RMSEA = 0.069, CFI = 0.928, TLI = 0.922, and SRMR = 0.042. IM remained positively associated with CPP (β = 0.472, *p* < 0.001) and LE (β = 0.481, *p* < 0.001), and CPP remained positively associated with LE (β = 0.387, *p* < 0.001). By contrast, the unique associations of EM with CPP (β = 0.085, *p* = 0.248) and LE (β = 0.085, *p* = 0.101) were not statistically significant. The indirect association between IM and LE through CPP remained significant [effect = 0.172, Boot SE = 0.038, 95% CI (0.104, 0.252)], whereas the corresponding EM indirect association was not significant [effect = 0.030, Boot SE = 0.028, 95% CI (−0.028, 0.081)].

**Figure 2:**
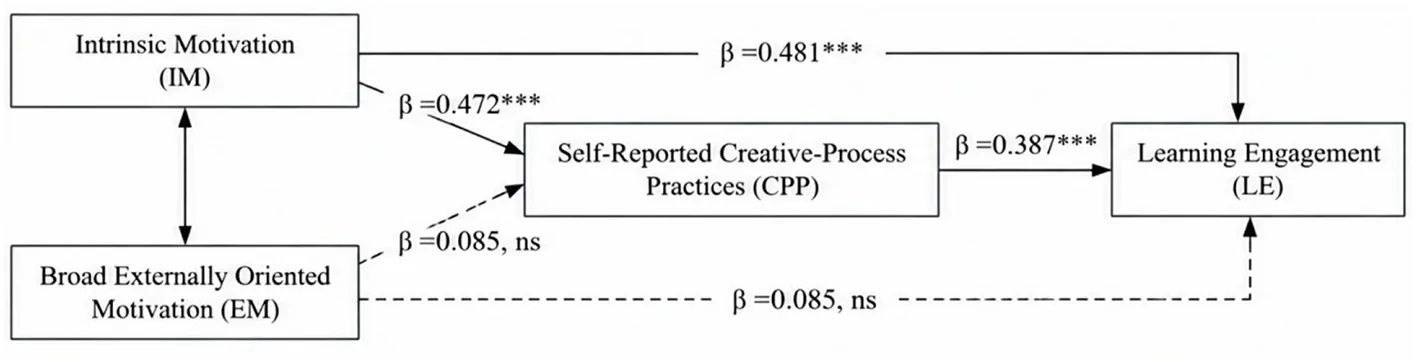
Simultaneous structural model with standardized path coefficients. Solid lines indicate significant paths, and dashed lines indicate nonsignificant paths. ***p < 0.001; ns = nonsignificant.

Across the three model specifications, the EM coefficients were smaller in the simultaneous model than in the EM-only model and were no longer statistically significant. By contrast, the IM coefficients remained statistically significant, while the CPP–LE association remained positive and statistically significant across all three specifications. Thus, in response to RQ2, the pattern of EM associations changed when IM was included in the same model, whereas the CPP–LE association was comparatively stable across specifications. These cross-model changes are interpreted descriptively rather than as formal statistical tests of coefficient differences.

## 6 Discussion

The findings showed a specification-dependent pattern. Both IM and EM were positively associated with CPP and LE when examined separately, but only IM retained statistically significant unique associations in the simultaneous model. CPP remained positively associated with LE across all three specifications.

### 6.1 Shared and unique associations of intrinsic and externally oriented motivation

The joint model therefore showed a clearer unique association for IM than for EM. Separate and simultaneous coefficients, however, answer different statistical questions: the separate EM model reflects its overall association with the outcomes, whereas the simultaneous model estimates the association unique to EM after its overlap with IM is taken into account. This distinction is consistent with evidence that different motivational orientations can coexist within students while showing different associations with educational outcomes (Howard et al., 2021; Cerasoli et al., 2014). Because no formal statistical test directly compared the IM and EM coefficients, however, the present results should not be interpreted as demonstrating that the IM coefficients were statistically larger than the EM coefficients.

The EM findings should also be interpreted in light of the breadth of the measure. The retained items capture broadly externally oriented motives related to achievement, recognition, and external outcomes but do not differentiate among external, introjected, identified, or integrated regulation. SDT emphasizes that externally oriented motivation varies substantially in its degree of internalization and psychological meaning (Ryan and Deci, 2020). Accordingly, the nonsignificant unique EM associations in the simultaneous model should not be generalized to specific forms of extrinsic regulation. The present quantitative data also cannot determine how students subjectively interpret goals related to recognition, assessment, exhibitions, or career preparation. Future qualitative or mixed-methods research could examine whether such goals are experienced as controlling demands, personally meaningful aspirations, or combinations of both.

### 6.2 Creative-process practices and learning engagement

The CPP–LE association was comparatively stable across model specifications. Because CPP and LE were measured as distinct constructs—reported creative-process practices vs. vigor, dedication, and absorption—this stability cannot be reduced to two measures of the same underlying construct (Schaufeli et al., 2002). Instead, it indicates that students who report greater participation in creative-process practices also tend to report stronger psychological engagement.

This relationship is particularly plausible in art education, where learning frequently unfolds through iterative making rather than through the acquisition of predetermined answers. Studio pedagogy typically requires students to generate possibilities, test ideas, revise work, respond to critique, and tolerate uncertainty during an extended creative process. Sawyer (2017) systematic review characterizes art studio learning as active, open-ended, participatory, and centered on students' engagement in authentic creative processes. Against this background, students who report greater involvement in experimentation and revision may also be more psychologically invested in their learning activities.

Nevertheless, the direction of this relationship cannot be established from the cross-sectional data. More engaged students may themselves be more willing to experiment, revise, and persist with alternative ideas, and reciprocal development is also plausible (Skinner et al., 2008). The indirect associations should therefore not be interpreted as evidence that CPP temporally mediates the relationship between motivation and engagement (Maxwell and Cole, 2007).

### 6.3 Contextual interpretation and alternative explanations

The coexistence of intrinsic and externally oriented motives is plausible in studio-based art learning, where open-ended creative work operates alongside assessment, critique, portfolio expectations, recognition, and professional goals (Sawyer, 2017; Amabile and Pratt, 2016). Students may therefore experience the same activity as intrinsically rewarding while also valuing its external outcomes.

The Chinese higher-education setting should, however, be interpreted cautiously. The present data show that intrinsic and externally oriented motives coexist among this sample of Chinese art students, but they do not establish that the observed motivational pattern is culturally distinctive. No cross-cultural comparison was conducted, and the findings should therefore be understood as evidence situated within Chinese higher art education rather than as evidence of a uniquely Chinese motivational structure. Whether the degree of motivational overlap or the pattern of EM associations differs across educational systems, disciplinary cultures, or assessment regimes remains an empirical question.

Several unmeasured factors may also contribute to the observed associations. Students with stronger perceived competence or creative self-efficacy, for example, may simultaneously report greater intrinsic interest, more willingness to experiment and revise, and stronger engagement. Autonomy-supportive instruction, the quality of studio feedback, peer climate, prior achievement, and students' broader achievement orientations could similarly shape more than one construct at the same time. Assessment requirements, workload, studio deadlines, and broader academic or professional pressure may likewise influence both students' motivation and their willingness to invest in creative-process practices and learning. SDT identifies autonomy, competence, and relatedness support as important conditions associated with more self-determined forms of motivation and positive educational functioning (Ryan and Deci, 2020), while longitudinal work on engagement emphasizes the role of both contextual support and students' self-perceptions in motivational dynamics (Skinner et al., 2008).

## 7 Theoretical and practical implications

### 7.1 Theoretical implications

The study contributes to motivational research by showing that correlated motivational orientations can yield different substantive interpretations depending on whether overall or unique associations are estimated. It also supports treating creative-process practices as a process-oriented correlate of learning engagement rather than as part of the engagement construct itself. Together, these findings underscore the importance of aligning construct definition with model specification when examining motivation and creative learning.

### 7.2 Practical implications

The findings suggest that art educators should avoid treating intrinsic and externally oriented motives as necessarily competing. Rather than attempting to eliminate external goals, teaching may help students connect achievement, recognition, and assessment demands with personally meaningful creative purposes and greater ownership of their work (Ryan and Deci, 2020).

The stable CPP-LE association also points to the potential value of making the creative process more visible in teaching. Process portfolios, staged critiques, iterative drafts, reflective journals, and feedback focused on experimentation and revision may provide students with clearer opportunities to engage in creative-process practices (Sawyer, 2017). These implications are theory-informed rather than intervention-tested, and should therefore be examined directly in future research.

## 8 Limitations and future research

Several limitations should be acknowledged. First, the cross-sectional design does not establish temporal ordering or causal mediation. The structural analyses were limited to the theoretically specified directional paths and did not estimate reciprocal or alternative causal-ordering models. Motivation, creative-process practices, and engagement may influence one another reciprocally, and longitudinal or cross-lagged designs are needed to examine these competing temporal possibilities more directly (Maxwell and Cole, 2007).

Second, all focal constructs were assessed through self-report within the same survey, so common method variance cannot be ruled out. Procedural features such as anonymity, confidentiality, and the use of different response formats across CPP and the other focal measures may reduce some response-consistency pressures but cannot eliminate this possibility. In particular, CPP represents students' reported creative-process practices rather than directly observed behavior. Future studies could combine self-reports with process portfolios, project records, observational measures, instructor ratings, temporally separated measurement, or other multi-source designs.

Third, the EM measure comprised only 4 retained items and captured a relatively broad set of achievement-, recognition-, and outcome-oriented motives without distinguishing among different forms of extrinsic regulation. The small number of retained items and their broad conceptual coverage may limit the comprehensiveness with which externally oriented motivation is represented. Future research should use more differentiated measures to examine whether external, introjected, identified, and integrated forms of regulation show different patterns when modeled alongside IM.

Fourth, the sample was purposively drawn from art programs in one Chinese province, with design students comprising the largest disciplinary group. The study was not designed to test subgroup differences by major, academic year, or weekly practice time, which were treated as sample characteristics rather than prespecified covariates. Generalization should therefore be cautious, and future multi-institutional studies should examine whether the observed associations vary across student subgroups and learning contexts.

The study also did not measure several potentially relevant individual and contextual factors, including perceived competence, creative self-efficacy, teacher support, feedback quality, assessment demands, workload, and academic or professional pressure. The reported associations should therefore not be interpreted as effects adjusted for these influences. Qualitative or mixed-methods research could further clarify how students interpret externally oriented goals such as recognition, assessment, exhibitions, and career preparation.

Finally, the linguistic adaptation should also be interpreted cautiously. Although professional translation, expert review, and pilot testing were used, no independent back-translation was conducted, and the 20-student pilot was intended primarily to identify wording and contextual issues rather than to provide a full psychometric validation of the adapted instrument.

## Data Availability

The raw data supporting the conclusions of this article will be made available by the authors, without undue reservation.
