# Supplementary material for: When externally oriented motivation appears to matter: shared and unique associations with creative-process practices and learning engagement among Chinese art students
Source: Front Psychol. 2026 Oct 21;17:1977647. doi: 10.3389/fpsyg.2026.1977647 (PMC13731107; doi:10.3389/fpsyg.2026.1977647)
Supplement: Supplementary file 1 [file Data_Sheet_1.pdf]

Supplementary Material 1: Additional Measurement Information

This supplementary material provides additional information on motivation-item screening and item-level measurement results for the final four-factor model. All reported results correspond to the final valid sample (N = 481) and the final 32-item measurement model comprising intrinsic motivation (IM), broad externally oriented motivation (EM), self-reported creative-process practices (CPP), and learning engagement (LE).

Table S1 Motivation items excluded from the focal measures and reasons for exclusion

Questionnaire item	Item content	Initial classification	Reason for exclusion	Empirical information
Item 12	Preference for less demanding art projects in order to ensure success	Initially included in EM item pool	Content reflected avoidance of challenge rather than the achievement- and recognition-oriented focus of the retained EM items	Standardized loading = 0.483
Item 13	Giving up and turning to a new work when outcomes do not meet expectations	Avoidance-oriented motivation	Conceptually distinct from both IM and broad EM	Loading on avoidance dimension = 0.777
Item 14	Avoiding difficult projects because of concern about failure	Avoidance-oriented motivation	Conceptually distinct from both IM and broad EM	Loading on avoidance dimension = 0.868
Item 15	Preferring to avoid challenging projects	Avoidance-oriented motivation	Conceptually distinct from both IM and broad EM	Loading on avoidance dimension = 0.714

Note. IItems 13 – 15 were excluded on conceptual rather than psychometric grounds. Their loadings on a separate avoidance-oriented dimension indicate that they represented a coherent motivational tendency outside the focal IM–EM distinction examined in the present study. Item 12 was excluded because its content reflected avoidance of challenge rather than the achievement- and recognition-oriented focus of the retained EM items, together with its comparatively weak standardized loading (0.483). These exclusions define the conceptual scope of the focal motivation measures and should not be interpreted as evidence that avoidance-oriented motivation is unimportant.

Table S2 Item wording and standardized CFA loadings for the final 32 retained items

Construct	Item	Observed variable / item wording	Standardized loading	SE
Intrinsic motivation	IM1	Engaging in art learning/creation makes me feel fulfilled.	0.818	0.016
	IM2	I engage in art learning/creation because it is highly enjoyable.	0.851	0.014
	IM3	I feel a strong sense of happiness when participating in art learning/creation.	0.870	0.012
	IM4	I feel a genuine need to understand artistic phenomena and artworks.	0.726	0.023
	IM5	I feel deeply joyful when learning something new in the field of art.	0.865	0.013
	IM6	For me, art learning/creation itself is a thoroughly pleasant experience.	0.897	0.010
	IM7	I love engaging in art learning/creation because it is inherently attractive.	0.876	0.012
Broad externally oriented motivation	EM1	I want to gain recognition from others by producing excellent artworks (e.g., winning awards, being selected for exhibitions).	0.901	0.011
	EM2	I want others to recognize me as a competent art learner/creator.	0.893	0.012
	EM3	I want to obtain external rewards (e.g., scholarships, competition prizes) through my artistic achievements.	0.818	0.017
	EM4	I want to leave my personal mark in the field of art.	0.847	0.015
Self-reported creative-process practices	CPP1	I attempt to utilize non-traditional materials or mediums for my artistic creation.	0.681	0.025
	CPP2	I integrate different artistic styles or movements into my own work.	0.759	0.020
	CPP3	I propose unique artistic insights or creative ideas during classes or group discussions.	0.745	0.021
	CPP4	I proactively seek sources of inspiration (e.g., visiting exhibitions, browsing art platforms) to stimulate my creation.	0.767	0.020
	CPP5	I adapt or re-create existing artworks to form novel expressions.	0.840	0.015
	CPP6	I attempt to solve technical or compositional problems encountered in my creation using	0.866	0.013

Construct	Item	Observed variable / item wording	Standardized loading	SE
		novel approaches.		
	CPP7	I record my fleeting creative thoughts (e.g., sketching, keeping an inspiration journal).	0.781	0.019
	CPP8	I proactively learn cross-disciplinary knowledge (e.g., technology, literature, philosophy) and apply it to my art creation.	0.791	0.018
	CPP9	I reflect critically on my own work and attempt to break through my previous creative paradigms.	0.847	0.014
	CPP10	I participate in artistic activities that require creative planning (e.g., themed creations, design competitions).	0.790	0.018
	CPP11	I deliberately experiment with non-conventional expressive techniques in my daily practice.	0.853	0.014
	CPP12	I transform my creative ideas into complete, finished artworks.	0.820	0.016
Learning engagement	LE1	When learning art, I feel full of vitality.	0.846	0.014
	LE2	I feel that the professional art knowledge I am learning is highly meaningful.	0.776	0.019
	LE3	When I am intensely focused on art creation or learning, time flies.	0.770	0.020
	LE4	While studying art, I feel highly energized.	0.851	0.014
	LE5	I am full of enthusiasm for my art learning.	0.899	0.010
	LE6	I feel deeply proud of studying art.	0.848	0.014
	LE7	When I am completely immersed in art learning, I forget everything around me.	0.774	0.019
	LE8	Learning art brings me immense joy.	0.881	0.011
	LE9	I get totally absorbed in my art learning or creation.	0.827	0.015

Note. IM = intrinsic motivation; EM = broad externally oriented motivation; CPP = self-reported creative-process practices; LE = learning engagement. Questionnaire items 12–15 were excluded from the final focal measures and are reported separately in Table S1. All standardized factor loadings were statistically significant at $p < 0.001$. The final measurement model contained 32 retained items.
